# Supplementary material for: The urban green carbon index (UGCI): A spatial framework for suggesting urban carbon management
Source: PLoS One. 2026 May 12;21(5):e0347782. doi: 10.1371/journal.pone.0347782 (PMC13166916; doi:10.1371/journal.pone.0347782)
Supplement: S1 File — (DOCX) [file pone.0347782.s001.docx]

**The urban green carbon index (UGCI): A spatial framework for suggesting urban carbon management**

**Supporting Information**

**Text S1 Roadside vegetation classification using the U-Net algorithm.**

We used the tree canopy cover map derived from Lee et al. (2024) as the roadside vegetation class layer in this study. The tree canopy cover map incorporated tree information from the high-resolution land cover map developed by Son et al. (2021), which was produced from airborne light detection and ranging (LiDAR) and 0.25 m RGB orthographic images using a U-Net architecture.

The RGB images were acquired in 2020 by the National Geographic Information Institute of South Korea (<https://www.ngii.go.kr/eng/main.do>), and the airborne LiDAR point cloud data were collected over the study site on September, 2020, using a Leica-Geosystems Terrain Mapper sensor (average point density ≈ 20.4 points m⁻²). From the point cloud, a digital surface model (DSM), digital terrain model (DTM), and intensity raster were generated at 0.25 m resolution using Esri ArcMap 10.1, and a height map was computed from the difference between DSM and DTM. These LiDAR-derived layers—DSM, DTM, height, and intensity—along with the RGB orthophotos, provided seven input layers offering fine-scale spectral and structural cues for urban vegetation mapping.

The classification used these seven input layers to identify five land-cover categories: trees, shrubs, grassland, building areas, and others. Trees and shrub labels were manually annotated, while the remaining classes were obtained from the subdivision land cover map provided by the Environmental Geographic Information Service of the Korean Ministry of Environment. The deep learning model followed the U-Net architecture, consisting of repeated blocks of convolutional layers, batch normalization, and ReLU activation functions. Each encoder block was connected to a max-polling layer, and each decoder block to an upsampling layer. Further details of the network configuration are provided in Son et al. (2021). Model training was performed on six 600 × 600 m subregions within the study site, representing diverse land-cover types such as forest, built-up, urban green, and agricultural areas. Validation was conducted on two independent 600 × 600 m test areas within the same city.

The U-Net model achieved an overall accuracy of 89.40%, and successfully identified 13.69% additional tree cover (including roadside vegetation) not represented in the existing subdivision land cover map. Remarkably, the binary classification accuracy for the “tree” class reached 96.29%. In this study, pixels classified as “tree” outside the three forest categories (broadleaf, coniferous, and mixed forest) in the Subdivision Land Cover Map were considered to represent roadside vegetation, following the approach of Lee et al. (2024).

**Text S2 Roadside vegetation carbon storage bias correction model.**

We applied the roadside vegetation carbon storage (VCS) bias correction model developed by Lee et al. (2024). To adjust the VCS estimates from Eqs 7 and 8 for roadside vegetation, we employed a machine-learning approach. Airborne LiDAR data-derived height and intensity maps, identical to those described in Supplementary Text S1, were generated at 0.25 m resolution. From these layers, zonal statics within the roadside vegetation areas (minimum, maximum, mean, and standard deviation for both, and percentiles for the height map) were computed for each 30 m analysis cell. To capture seasonal canopy conditions, Sentinel-2 Normalized Difference Vegetation Index (NDVI) was derived from red and near-infrared bands on June 21 and October 29, 2020 at 10 m resolution. NDVI values were summarized by the same 30 m grid structures with its statistics (minimum, maximum, mean, and standard deviation).

Field-based VCS reference data were obtained from 69 roadside vegetation plots (30 × 30 m) surveyed in 2021. For each plot, the diameter at breast height (DBH) of individual trees was measured using terrestrial LiDAR point clouds (Leica BLK360), and species-specific allometric equations were applied to compute per-cell carbon storage (Eq 8). For intersection grid boundaries, inclusion ratios were determined using terrestrial LiDAR point clouds to minimize edge bias.

The bias (VCS from Eq 8 minus VCS from Eq 7) was modeled using supervised machine learning with the LiDAR and Sentinel-2 predictors described above. The final bias-corrected VCS was obtained by adding the predicted bias from the machine learning model to the original VCS from Eq 7. Candidate algorithms included Random Forest, XGBoost, and Support Vector Regression. Model selection was based on 10-fold cross validation, using R^2^ and root mean squared error (RMSE) as evaluation metrics. Hyperparameter tuning was conducted using the “GridSearchCV” function in Python. The Random Forest model achieved the best performance, with an average R^2^ of 0.884 and RMSE of 0.432 tC/900m^2^. This model was therefore used to correct VCS estimates from Eq 7 across the entire study area. Further details and validation results are provided in Lee et al. (2024).

**Text S3 Modelling ecosystem respiration (Reco).**

The model estimating ecosystem respiration (R_eco_) used a temperature-response function that incorporates short-term carbon assimilation history, reflecting the empirically observed lag between photosynthesis and respiration. The selected formulation (Eq S1) was:

$R_{eco}=(a+b\times{GPP}_{acc})\times exp(c\times\frac{T_{air}}{\max\left( T_{air} \right)})$ (Equation S1)

where *R_eco_* is the ecosystem respiration (μmol CO_2_ m⁻² s⁻¹), *a*, *b*, and *c* are empirical parameters, *GPP_acc_*​ is the accumulated gross primary productivity (μmol CO_2_ m⁻² s⁻¹), and *T_air_* is the air temperature (℃).

The model parameters (a = 1.0, b = 0.2, c = 1.156) and the 7-day window for accumulated gross primary productivity (GPP) were determined empirically using the FLUXNET2015 eddy covariance dataset (hourly R_eco_ from 1995-2014) and associated meteorological variables. The fitting employed a non-linear least squares optimization (Levenberg-Marquardt algorithm) with parameter initialization ranges *a*, *b*, and *c* ∈ [0, 2].

The model explicitly compared several commonly used temperature-based respiration formulations, including linear temperature dependence (linT), exponential temperature response (ExpT), and EVI-modulated exponential formulation (EVIexpT) (Mahadevan et al., 2008; Yan et al., 2015). The formulation in Eq S1 was selected because it provided comparable or superior performance to these alternatives while avoiding plant-functional-type-specific parameterization.

Calibration and performance evaluation of the R_eco_ model were performed in two steps: (i) internal calibration using FLUXNET2015 and (ii) external validation using eddy-covariance tower observation. Model parameters were first optimized using the full FLUXNET2015 half-hourly dataset, restricted to site-years that passed quality filtering. Validation statistics (R², RMSE, mean-ratio, and variance-ratio) were computed for each site-year and then averaged to obtain site-level metrics. The calibrated R_eco_ model was subsequently applied to an independent eddy-covariance site (2016–2018) using only remotely derived meteorological and satellite inputs. The modeled hourly R_eco_ was compared against tower-observed fluxes, confirming that the formulation correctly reproduced seasonal and diurnal patterns and outperformed all comparison models when combined into NEP simulation.

**Text S4 Comparison between UGCI and an existing index.**

To examine the applicability of the UGCI in urban environments, we compared both the statistical and spatial distributions of the UGCI with those of the carbon sequestration potential index (CSPI) developed by Pascual et al. (2020). The CSPI was originally designed to identify suitable areas for afforestation and reforestation management across six Hawaiian Islands using 30 m resolution datasets. The calculation of the CSPI is as follows:

$CSPI=GPP \times\left( ACD \right)^{-1}\times(1-FC)$ (Equation S2)

where GPP (gross primary production) (kgC m^-2^ yr^-1^), ACD (aboveground carbon density) (kgC m^-2^), and FC (Forest coverage) (%).

We used the GPP (tC ha^-1^ yr^-1^) and vegetation C storage (tC ha^-1^) developed in this study to calculate the CSPI. To adapt the CSPI for an urban context, the forest coverage term in each grid was replaced with green-space coverage. After calculating the CSPI, the results were min-max normalized, following the same procedure used for UGCI visualization, to enable a direct comparison between CSPI and UGCI.

Supplement figures


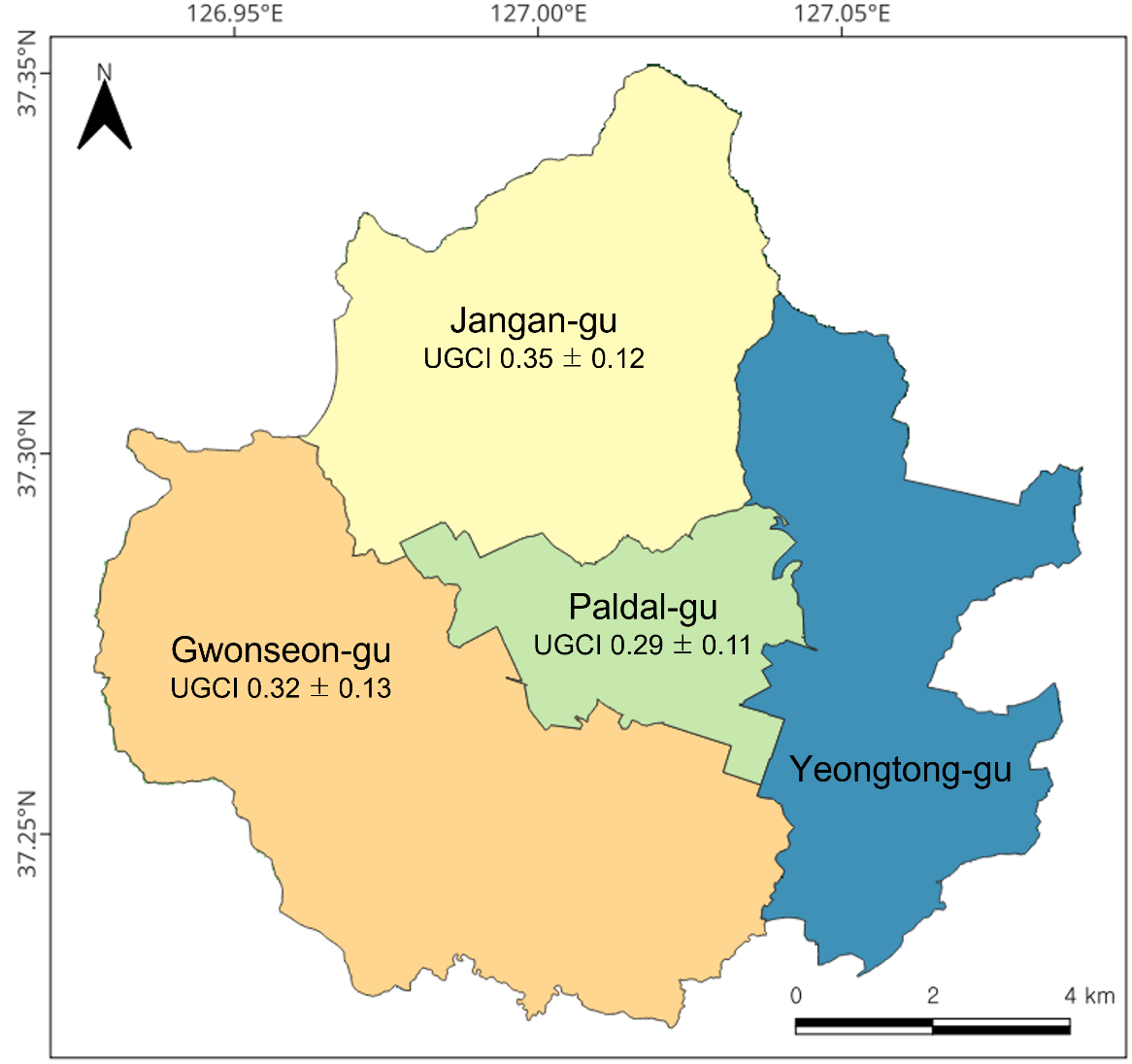


Fig S1 Administrative districts of the study site and their average UGCI values.


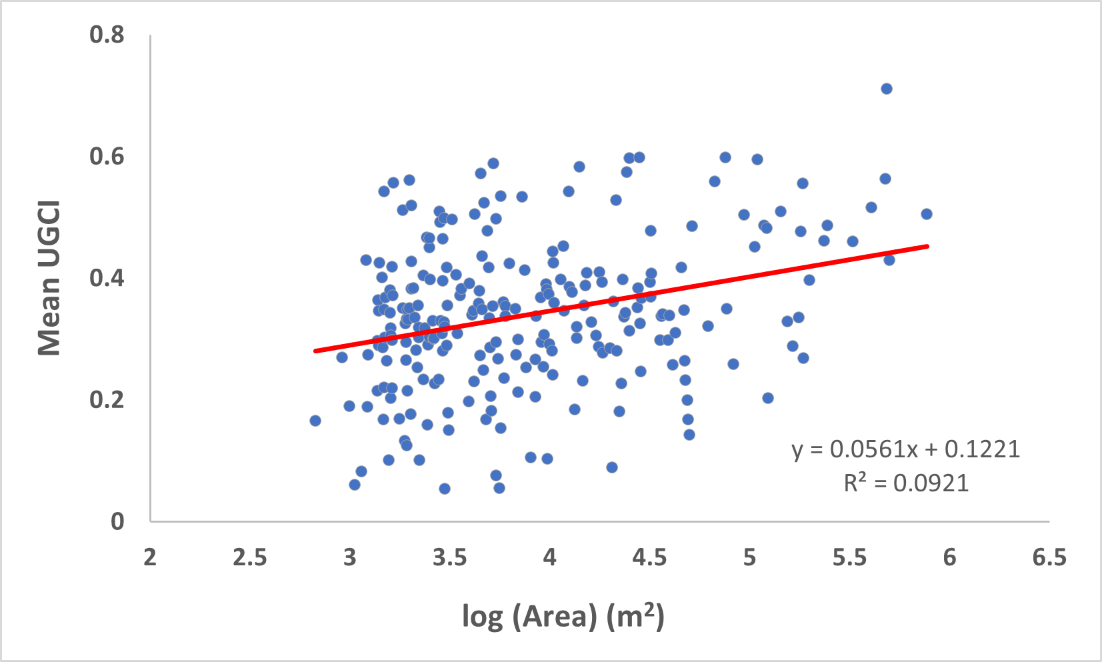


Fig S2 The correlation between the areas of green patches in urban parks (logarithmic scale) and their mean UGCI. Blue points represent individual green patches within urban parks, and the solid red line denotes the fitted linear regression.


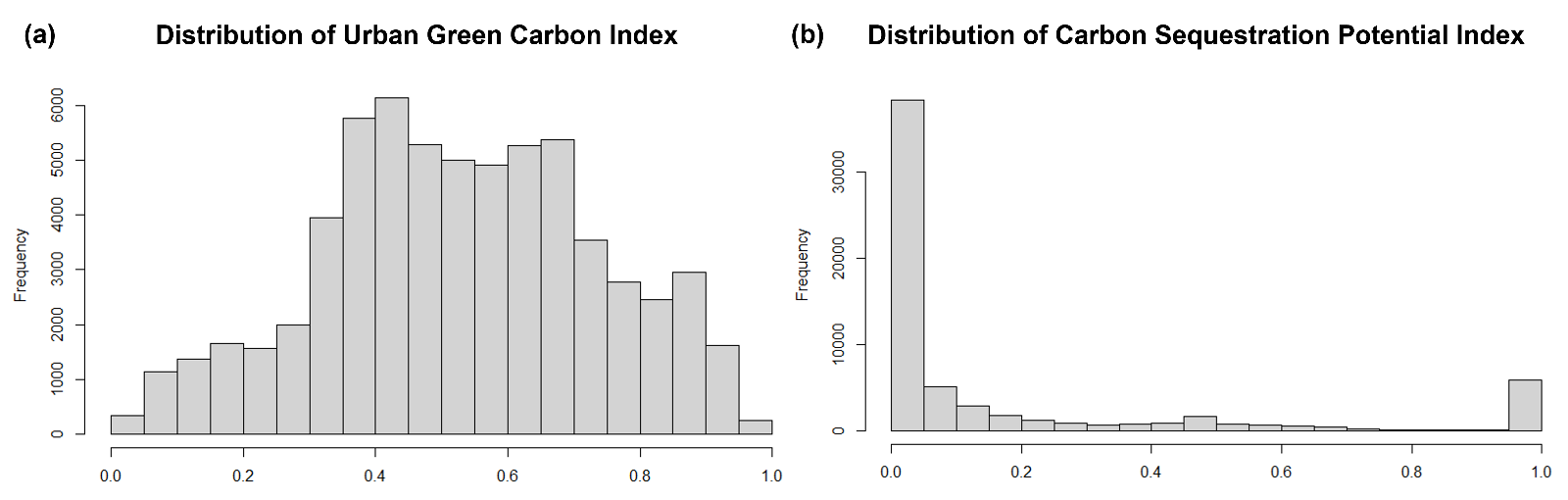


Fig S3 Data distribution of urban green carbon index (UGCI) and carbon sequestration potential index (CSPI).

Supplement Table

Table S1 Optimized model hyperparameters and model performance metrics for soil silt and clay content mapping.

| Algorithm | Optimized hyperparameter values | R^2^ | RMSE (%) |
| --- | --- | --- | --- |
| Random forest | mtry = 12, node size = 5 | 0.58 | 10.34 |
| Artificial neural network | hidden 1 = 90, hidden 2 = 81, rate = 0.0094 | 0.64 | 10.16 |
| Light gradient boosting model | learning rate = 0.01, num leaves = 21, min data in leaf = 14, feature fraction = 0.7247, bagging fraction = 0.8629 | **0.84** | **10.33** |

**References**

Lee Y, Son B, Im J, Zhen Z, Quackenbush LJ. Two-step carbon storage estimation in human settlements in urban areas using airborne LiDAR and Sentinel-2 data based on machine learning. Urban For Urban Green. 2024 Feb;128239.

Mahadevan P, Wofsy SC, Matross DM, Xiao X, Dunn AL, Lin JC, et al. A satellite-based biosphere parameterization for net ecosystem CO2 exchange: Vegetation Photosynthesis and Respiration Model (VPRM). Glob Biogeochem CYCLES. 2008 Apr 12;22:GB2005.

Pascual A, Giardina CP, Selmants PC, Laramee LJ, Asner GP. A new remote sensing-based carbon sequestration potential index (CSPI): A tool to support land carbon management. For Ecol Manag. 2021 Aug;494:119343.

Son B, Lee Y, Im J. Classification of Urban Green Space Using Airborne LiDAR and RGB Ortho Imagery Based on Deep Learning. J Korean Assoc Geogr Inf Stud. 2021 Sep 30;24(3):83–98.

Yan W, Hu Z, Zhao Y, Zhang X, Fan Y, Shi P, et al. Modeling Net Ecosystem Carbon Exchange of Alpine Grasslands with a Satellite-Driven Model. Piao S, editor. PLOS ONE. 2015 Apr 7;10(4):e0122486.
