# Supplementary material for: The urban green carbon index (UGCI): A spatial framework for suggesting urban carbon management
Source: PLoS One. 2026 May 12;21(5):e0347782. doi: 10.1371/journal.pone.0347782 (PMC13166916; doi:10.1371/journal.pone.0347782)
Supplement: Attachment — Submitted filename: Response to reviewer_UGCI_final.docx [file pone.0347782.s003.docx]

**Response to Reviewers [PONE-D-25-21587]**

Dear Dr. Tianheng Shu, Academic editor:

Thank you for the opportunity to revise our manuscript, ID: PONE-D-25-21587, titled “A Comprehensive Decision-making Framework for Prioritizing Carbon Management in Complex Urban Landscape .” We are grateful to you and the esteemed reviewers for the time and effort dedicated to providing insightful and constructive feedback. The comments have been invaluable in helping us strengthen the manuscript significantly.

We have thoroughly addressed all the points raised by the reviewers and have revised the manuscript accordingly.

The main changes incorporated into the revised manuscript are summarized below:

- **Conceptual refinement of the index:** Following a critical suggestion from Reviewer #2, we have renamed the "Carbon Management Prioritizing Index (CMPI)" to the "Urban Green Carbon Index (UGCI)." This change more accurately reflects the index's function as a diagnostic tool for assessing carbon status, distinct from a full cost-benefit or opportunity-cost analysis, thereby clarifying the manuscript's core contribution. Accordingly, we changed the title of the manuscript from ‘*A Comprehensive Decision-making Framework for Prioritizing Carbon Management in Complex Urban Landscape*’ to ‘*The urban green carbon index (UGCI): A spatial framework for suggesting urban carbon management.*’
- **Strengthened methodological justification:** We have provided more detailed explanations for key methodological decisions. This includes a clearer rationale for selecting the 30 m grid size, justification for the choice of input variables for our GPP/Reco models (as suggested by Reviewer #5), and a more transparent description of the data resampling and processing techniques (as requested by Reviewer #4).
- **Enhanced validation and contextualization:** To address comments regarding the validation of our models and the novelty of our index (Reviewers #2, #4, and #5), we have expanded our discussion. We now include a direct quantitative comparison of the UGCI with an existing metric, the Carbon Sequestration Potential Index (CSPI), to better situate our work within the current literature and highlight its unique contributions to urban carbon management.
- **Refinement of interpretations and claims:** Based on the feedback concerning potential logical fallacies (Reviewers #2 and #4), we have carefully revised our interpretations. For instance, we replaced ambiguous terms like "degraded" with more precise descriptions such as "under poor condition" and have moderated our claims where correlations were weak, ensuring our conclusions are robustly supported by the evidence presented.
- **Improved reproducibility and clarity:** In response to requests for greater detail (Reviewers #4 and #5), we have added specific parameters for equations, elaborated on model validation procedures, and moved detailed methodological descriptions to the supplementary materials to ensure clarity and reproducibility without disrupting the flow of the main text. The entire manuscript has also undergone a thorough proofread to improve language and readability.

Here, we provide responses following the comments by the reviewers.

**Reviewer #1:**

***This paper presented a spatial decision-making framework for the urban carbon sequestration management based on a novel Carbon Management Prioritizing Index (CMPI) and the ternary plots. The CMPI integrates the spatial data on vegetation C storage, soil C storage, net C uptake, and soil C storage potential and complete the diagnosis procedure and the ternary plots implements site-specific C management strategies. This framework was applied to a case study in Suwon, South Korea. Generally, this research is interesting and the framework can be applicable to urban C management. The writing-up of this manuscript is generally acceptable, but some clarifications and improvements are still necessary. My particular comments are as follows.***

Response: Thank you for your positive and encouraging review. We sincerely appreciate the thoughtful and constructive feedback. Your suggestions have significantly strengthened the manuscript, and we have revised the article to incorporate them. My detailed responses to each of your comments are outlined below.

***1. What is the minimum grid size for a landscape simulation? This one should be discussed in details because it concerns the balance between computation load and accuracy.***

Response: We appreciate this valuable comment. In our case study, the spatial resolution of the four components constituting the framework was unified to 30 m. This resolution was chosen by balancing the minimum available spatial resolution of the input datasets and the spatial granularity necessary for urban-scale analysis. Although a 1 m land cover map was available for assessing vegetation C storage, the other components lacked data at such a fine scale. Soil C storage was derived from the GSOCmap at 1 km resolution, and net C uptake component and soil silt and clay content were modeled at 250 m and 30 m resolution, respectively, reflecting the finest spatial resolutions of the available input dataset. Table 1 describes detailed information on input datasets. We have added this explanation to Section 2.2 Case study to clarify the rationale for selecting a 30 m grid.

- Revised Text (Lines 190-193): In this case study, the data collection was conducted for the year 2020. All spatial data layers were resampled to a 30 m resolution, balancing the finest available resolution of input datasets with the spatial granularity required for urban-scale analysis.

***2. All equations should have their number.***

Response: Thanks for pointing it out. We have revised the manuscript so that all equations are now numbered consistently and can be clearly referenced in the text (Lines 124, 131, 143, 146, 150, 167, 228, 246, 288-289, 309).

***3. Is whether ‘Section 2.2 Case Study’ here suitable or not? Is the framework associated with case study? My understanding is that Case study only demonstrates the application of this framework.***

Response: We appreciate the reviewer’s constructive comment. The case study section was included not as an independent example, but as an essential process to demonstrate the applicability of the proposed framework in an actual urban environment. The framework itself is conceptual and methodological, and its practical relevance can only be evaluated through real-world implementation. Therefore, the case study serves as a proof of concept, showing how the framework operates under real urban conditions, including data acquisition and generation, possible analysis, and interpretation. For this reason, we maintained it as a dedicated section. To clarify the role of the case study within this research, we have added the purpose of implementing this case study at the beginning of Section 2.2 Case Study.

- Original Text (Lines 187-190): To validate the effectiveness of the proposed framework, we applied it to Suwon, a representative metropolitan city in South Korea. Suwon (37°17'28" N, 127°0'32" E) is the third most densely populated city in the country, with a population density of 9,884 persons km⁻² as of 2024 (Korean Ministry of the Interior and Safety).
- Revised Text (Lines 181-185): Through the case study, we demonstrate the applicability of the proposed framework and evaluate the practical availability of UGCI in an actual urban environment. Our study site was Suwon (37°17'28" N, 127°00'32" E), a representative metropolitan city in South Korea with a population density of 9,884 persons km⁻² as of 2024 (Korean Ministry of the Interior and Safety).

***4. English and presentations should be significantly improved for clear and correct statements or expressions***

Response: We sincerely appreciate the reviewer’s feedback regarding language clarity. The entire manuscript has been thoroughly revised to improve readability and precision in expression. All sentences were carefully re-checked using both professional editing tools and manual proofreading to ensure grammatical accuracy and clarity.

***Reviewer #2:***

***This study presents a spatially explicit framework for diagnosing and prioritizing urban carbon management using a newly developed Carbon Management Prioritizing Index (CMPI) and ternary plots. Overall, the study is timely and policy-relevant. I particularly appreciate the integration of both vegetation and soil carbon components and the use of high-resolution data in a highly urbanized context. However, several issues need to be addressed to improve the scientific rigor, policy applicability, and clarity of the manuscript. I recommend major revision before further consideration for publication.***

Response: Thank you for your positive and encouraging feedback on the manuscript. We sincerely appreciate the time you took to provide such thoughtful and constructive suggestions. The manuscript has been revised to incorporate these changes, which we believe have significantly improved the article. Please find our detailed responses to each of your comments outlined below.

***1. Silt and clay content is an intrinsic parent-material property that cannot be manipulated by urban green-space management on policy-relevant timescales. Thus, I think that including it in a “management prioritisation index” (CMPI) is conceptually invalid. Moreover, the use of silt and clay content alone as a proxy for soil carbon potential is overly simplistic and may overlook key edaphic factors (e.g., pH, bulk density, microbial activity). In addition, due to its high spatial heterogeneity at the fine scale, silt and clay content receives the highest weight (>0.4), while vegetation C stock, the only component directly manageable by urban forestry, is down-weighted. The outcome is a mathematically tractable but ecologically meaningless index.***

Response: We appreciate the reviewer’s valuable and constructive comments. We fully agree that soil texture is an intrinsic property that is not easily altered and may not always serve as a direct management lever in urban green policy. However, it is important to note that our study encompasses urban green spaces, which were not represented by single and undisturbed ecosystems such as natural forests. Most urban soils are constructed or restored, typically consisting of sandy materials used as fill. Under such conditions, the composition of silt and clay can be modified or selected during construction and restoration processes.

We also acknowledge that silt and clay content alone cannot represent all edaphic conditions. Nevertheless, soil texture is known as a primary parameter governing key physical and biological properties such as bulk density, aeration, and microbial habitat, which are fundamental to C protection and turnover. Because pH does not exhibit a direct relationship with C status, we did not include it in our index. These points have been incorporated into Section 2.2.4 Soil C storage potential: Silt and clay content.

- Original Text (Lines 308-310): Because the soil silt and clay content critically influences soil C storage potential by enhancing soil’s capacity to stabilize C [39,40], we considered it as an indicator of soil C storage potential.
- Revised Text (Lines 321-327): Given that soil silt and clay content play a critical role in stabilizing C [39,40], it was used as an indicator of soil C storage potential. While silt and clay content is intrinsic and relatively stable in natural soils, urban soils—particularly those in parks and roadside vegetation—are often reconstructed and sand-dominated, allowing urban soil texture to be modified during construction. Other edaphic factors, such as pH and microbial activity, were not incorporated because their direct relationships with soil C storage potential remain uncertain.

In addition, the weight for silt and clay content component was the lowest of 0.08, we have added the exact number in Section 3.3 Site-specific C management strategies and policy implications.

- Original Text (Lines 443-445): Since soil silt and clay content exhibited minimal variability, it was excluded from the ternary plots, focusing on vegetation C storage, soil C storage, and net C uptake.
- Revised Text (Lines 459-461): Among the components of the UGCI, the soil silt and clay content had the lowest weighting factor (0.08), indicating the smallest contribution to the overall variation of the index; therefore, it was excluded from the ternary plot analysis.

***2. I am confused that high-biomass forests receive low CMPI scores and are consequently ranked as “low priority”, whereas low-biomass roadside plantings receive high CMPI scores. The authors implicitly assume that marginal C gains are automatically larger where current stocks are low, but supply no cost–benefit or marginal abatement cost curve to support this assumption. In fact, mature forests often provide the largest, cheapest avoided emissions simply by conserving existing carbon; ignoring this principle undermines the entire prioritisation logic.***

Response: We appreciate your critical view. We recognize that the term ‘Carbon Management Prioritizing Index’ could be misleading, since management priority may imply the inclusion of cost-benefit or opportunity cost analyses. However, our research intention was to provide spatially explicit information on vegetation and soil C status prior to determining C management priorities. Therefore, we revised the name of index from CMPI to urban green carbon index (UGCI) throughout the manuscript. As we acknowledge the limitation of not incorporating policy implementation pathways and cost–benefit analysis, we included this limitation in Section 2.1 where UGCI was first introduced and in Section 3.5. Limitation and future study.

- Original Text (Lines 106-108): This approach ensures that proposed management actions are both locally appropriate and practically effective for enhancing urban C sequestration capacity.
- Revised Text (Lines 105-107): The proposed strategies do not incorporate cost-benefit or opportunity-cost analyses; these limitations and the corresponding directions for future research are discussed separately in the discussion section.
  (Lines 534 - 542) This study had several clear limitations that can be addressed in future research. First, the UGCI was currently focused on C enhancement and did not integrate policy implementation pathways or cost-benefit considerations. Because its primary role was to provide a scientifically grounded, spatially explicit diagnosis of low-carbon areas and management directions, economic assessments were intentionally kept external to the index. Future studies could link the UGCI with additional decision layers, such as cost-effectiveness, marginal abatement cost, life-cycle assessment, or budgeting, through multi-criteria decision analysis. This would enhance the applicability of the index while preserving flexibility to local financial capacities and policy priorities.

The assumption pointed out by the reviewer that areas with low carbon stocks may exhibit high marginal carbon gains is a concept commonly applied in the context of urban and anthropogenically managed green spaces. For instance, Pascual et al. (2020) developed the Carbon Sequestration Potential Index (CSPI) to identify priority areas for afforestation and reforestation, assigning higher index values to regions with lower forest cover or lower photosynthetic efficiency per unit biomass. Georgiou et al. (2022) demonstrated that soils with a greater deficit from their carbon-saturation capacity accumulated carbon more rapidly through management interventions than soils approaching saturation. These studies collectively support the rationale underlying our assumption. Nevertheless, we fully agree with the reviewer that the conservation of mature forests is a critical management strategy from the perspective of avoided carbon emissions. However, low index values do not necessarily indicate that management is unnecessary; rather, it can lead to conservation-oriented management strategies. We have added this clarification to the Section 3.3. Site-specific C management strategies and policy implications.

- Original Text (Lines 442-445): Figure 7 presents ternary plots illustrating grids classified as ‘high’ and ‘extremely high’ CMPI levels, differentiated by land cover type. Since soil silt and clay content exhibited minimal variability, it was excluded from the ternary plots, focusing on vegetation C storage, soil C storage, and net C uptake.
- Revised Text (Lines 458-465): Fig 6 presents ternary plots illustrating grids classified as ‘low’ and ‘extremely low’ UGCI levels, differentiated by land cover type. Among the components of the UGCI, the soil silt and clay content had the lowest weighting factor (0.08), indicating the smallest contribution to the overall variation of the index; therefore, it was excluded from the ternary plot analysis. While our analysis here concentrates on the ‘low’ and ‘extremely low’ UGCI levels, it is important to note that a high UGCI does not imply the absence of management needs. Conservation-oriented strategies aimed at protecting existing C stocks and avoiding disturbances may be more appropriate than aggressive intervention in such areas.

Reference:

1. Pascual A, Giardina CP, Selmants PC, Laramee LJ, Asner GP. A new remote sensing-based carbon sequestration potential index (CSPI): A tool to support land carbon management. Forest Ecology and Management. 2021 Aug;494:119343.
2. Georgiou K, Jackson RB, Vindušková O, Abramoff RZ, Ahlström A, Feng W, et al. Global stocks and capacity of mineral-associated soil organic carbon. Nat Commun. 2022 Jul 1;13(1):3797.

***3. A unit loss in vegetation C is compensated by an equivalent gain in soil C or NEP. In reality vegetation removal often accelerates soil C loss, producing positive feedbacks. The linear model ignores the inter-process coupling and nonlinear thresholds and synergies, which is a primary error in ecological modeling.***

Response: We appreciate the reviewer’s valid comment. We fully agree that vegetation-soil-NEP interactions are inherently nonlinear, and that vegetation removal in natural ecosystems can accelerate soil carbon loss through positive feedback mechanisms. However, the UGCI was not designed as a mechanistic ecological model but rather as an index for management. The weighted-mean formula of UGCI should therefore not be interpreted as representing a linear trade-off, but as a combination of independent indicators of current carbon storage and carbon sequestration potential.

In addition, vegetation loss in urban green management typically occurs through pruning or thinning, rather than complete biomass removal, and thus is less likely to induce significant SOC losses compared to natural ecosystem disturbances. Nevertheless, we acknowledge that the current UGCI framework does not explicitly incorporate ecological inter-process coupling or nonlinear thresholds, and this limitation has been stated in Section 3.5. Limitation and future study.

- Revised Text (Lines 543-546): Second, the UGCI framework did not explicitly account for ecological interactions or nonlinear feedback among its constituent components. Once these interrelationships are quantitatively understood, incorporating them into the index could improve its ability to represent complex ecosystem processes and capture spatiotemporal variability in C dynamics.

***4. Soil C data are taken from the 1 km Global Soil Organic Carbon map (GSOC v1.6) and resampled to 30 m with bilinear interpolation. Interpolation cannot create new information; the “30 m” soil layer still carries 1 km effective support. The validation R2 = 0.84 derives from 121 national soil samples that were never designed to test 30 m spatial accuracy. Using this as evidence of high-resolution reliability is misleading.***

Response: We appreciate the reviewer’s careful observation. We recognize that the original order of sentences may have caused the meaning of ‘validation’ to be misleading. We intended not to claim high-resolution accuracy, but rather to show that the GSOC v1.6 product is sufficiently reliable for use within our framework at its original 1 km resolution. To avoid confusion, we have revised the text to clarify the sequence as follows:

- Original Text (Lines 260-269): Soil C storage data were obtained from the Global Soil Organic Carbon (GSOC) map (version 1.6.1, 1 km spatial resolution) [31,32]. To unify the spatial resolution with other datasets, we resampled the map to a 30 m resolution using bilinear interpolation method. To ensure data reliability and suitability for our analysis, we validated the GSOC map using soil C storage measurements derived from 121 legacy soil samples collected from diverse land-cover types across South Korea during 2020–2024 by the Kyung Hee University Carbon Neutrality Technology Center [33,34]. The validation showed a high coefficient of determination (R2 = 0.84), confirming that the GSOC data were sufficiently accurate and reliable for integration into our study framework. Additional details on data accuracy and uncertainty are available in the GSOC map v1.6 Technical Report [31].
- Revised Text (Lines 262-271): Soil C storage data were obtained from the Global Soil Organic Carbon (GSOC) map (version 1.6.1, 1 km spatial resolution) [31,32]. To ensure data reliability and suitability for our analysis, we validated the GSOC map using soil C storage measurements derived from 121 legacy soil samples collected from diverse land-cover types across South Korea during 2020–2024 by the Kyung Hee University Carbon Neutrality Technology Center [33,34]. The validation showed a high coefficient of determination (R2 = 0.84), confirming that the reliability of the product for our purpose. Additional details on data accuracy and uncertainty are available in the GSOC map v1.6 Technical Report [31]. To unify the spatial resolution with other datasets, we resampled the map to a 30 m resolution using a bilinear interpolation method, preserving its continuous gradient.

***5. GPP and Reco models are calibrated with Mt. Taehwa eddy-covariance data (a peri-natural forest on rugged terrain 400 m a.s.l.) and applied to inner-city grids where surface energy balance, aerosol load, irrigation, and heat-island effects differ drastically. No site-specific validation is provided for Suwon’s urban land covers. Systematic over-estimation of NEP is highly probable.***

Response: We acknowledge the reviewer’s valid comment that the model validation may have been overestimated since it was conducted only for the Mt. Taehwa. Unfortunately, no eddy covariance tower was available in Suwon, our main study area, and thus direct validation of NEP for Suwon was not possible. Mt. Taehwa EC tower, which has been continuously operating since 2016, was the only available dataset for this purpose. Although Mt. Taehwa eddy-covariance tower is located outside our study site, the surrounding environment is characterized by urban green space rather than a purely natural forest, as it is adjacent to roads and other residential infrastructure (Figure 1).

Figure 1 The location of Mt. Taehwa which is located nearby the residential infrastructure.

Nevertheless, we fully agree that the reviewer’s point is crucial for accurate NEP estimation. To further examine this issue, we compared GPP and Reco data from Mt. Taehwa with those from urban green sites (Mt. Nam and Changgyeong Palace in Seoul) that have been operated since 2024 (unpublished data). Across all urban sites, the annual integrated GPP was approximately 0.7 times than that of Mt. Taehwa, while Reco was about 1.0-1.2 times higher. The temporal changing patterns of both GPP and Reco were consistent among the three sites. Despite the differences in absolute values, NEP was incorporated into the index after normalization, and we considered this level of discrepancy acceptable within the scope of our framework. As the number and duration of EC tower observations in urban green spaces continue to increase, further improvement of NEP estimation models will be possible in future research. This was revised in Section 2.2.3.

- Original Text (Lines 291-293): The GPP model was trained using the FLUXNET2015 dataset [36] and validated against local flux tower measurements at Mt. Taehwa in South Korea (37°18'15" N, 127°18'50" E), achieving a R2 of 0.76 and a root mean square error (RMSE) of 0.41 μmol CO_2_ m^-2^ s^-1^.
- Revised Text (Lines 298-303): The GPP model was trained using the FLUXNET2015 dataset [36] and validated against local flux tower measurements at Mt. Taehwa in South Korea (37°18'15" N, 127°18'50" E), achieving an R^2^ of 0.76 and an RMSE of 0.41 μmol CO2 m^-2^ s^-1^. Although Mt. Taehwa is located outside our study site, the temporal variation patterns of its GPP and R_eco_ data were consistent with those observed at the eddy covariance towers in Mt. Nam and Changgyeong Palace, representative urban green areas in Seoul.

***6. Roadside plots cluster near the centre of the ternary diagram and are declared “multi-dimensionally degraded”. Given their young age, small size and engineered substrates these plots are expected to start with low biomass; without a temporal baseline the authors cannot demonstrate a decline (degradation). The interpretation is therefore a logical fallacy.***

Response: We acknowledge that the ternary plot does not include any information on temporal change, as UGCI was calculated from single-year data (2020). Therefore, we agree that the term ‘degraded’ was inappropriate in this context. All instances of ‘degraded’ have been replaced with ‘under poor condition’ (Lines 71, 80, 91, 99, 153, 163, 421).

***7. Forest edges exhibit 34 % higher CMPI than interiors (p < 0.001). The authors immediately prescribe “buffer zones”, yet provide no evidence that the CMPI difference corresponds to measurable biomass loss or soil C reduction.***

Response: We appreciate the reviewer’s valuable comment. We acknowledge that the rationale for defining the buffer zone was not clearly presented. Therefore, we have clarified that the low UGCI values observed near forest edges are mainly attributed to lower soil C storage, and revised the corresponding text as follows:

- Original Text:
  (Lines 32-34) Based on this analysis, prioritized management strategies included establishing buffer zones at forest edges, mitigating soil compaction in urban parks, and implementing multi-layered planting in roadside vegetation.
  (Lines 446-451) In forests, all grids classified as ‘high’ CMPI were situated at edge areas, emphasizing targeted edge-focused management. The points concentrated around the soil C storage vertex highlight soil C as the primary driver of degradation. Management practices in forest edges should thus prioritize creating buffer zones adjacent to impervious surfaces to moderate environmental stresses, such as high temperatures and low humidity, as well as maintaining litter layers and improving soil structure to reduce C losses [69,70].
- Revised Text:
  (Lines 32-34) Based on the limited components revealed by the ternary analysis, tailored management practices—such as surface litter retention, soil decompaction, and multi-layered planting—were proposed to improve the weaker C components.
  (Lines 466-472) In forests, all grids classified as ‘low’ UGCI were spatially situated at edge areas, emphasizing the need for edge-focused management. These edge grids consistently exhibited the lowest scores for soil C storage, indicating that management efforts should primarily focus on enhancing soil C stability. Because forest-edge soils are more exposed to higher temperatures and direct sunlight, leading to accelerated decomposition [53,67], promoting understory vegetation and maintaining surface litter can help minimize soil exposure and sustain soil moisture.

***8. Urban parks show a weak negative correlation (r = –0.27) between patch size and CMPI. The authors conclude that “smaller patches need intervention”. However, small parks are often sport fields or playgrounds with intentionally low biomass; again the paper confers a management necessity onto a land-use pattern that is by design.***

Response: We acknowledge the reviewer’s feedback. As the reviewer pointed out, due to the weak correlation and the fact that management interventions are not feasible in certain parks, we revised the discussion to the following sentence.

- Original Text (Lines 428-434): Additionally, CMPI was inversely related to patch size in urban parks, indicating smaller patches generally had higher CMPI values (r = -0.27, p < 0.001; Fig. 6). Larger patches inherently support more complex ecological structures, higher vegetation density, and greater soil C inputs, leading to improved C sequestration capacity [65–68]. Thus, CMPI effectively reflects the ecological and landscape characteristics that influence urban green spaces’ C sequestration potential. Overall, our results confirm that CMPI is a robust diagnostic tool for identifying and prioritizing urban green spaces requiring targeted management interventions.
- Revised Text (Lines 447-455): Additionally, the correlation between the areas of green patches in urban parks (logarithmic scale) and their mean UGCI was very weak (r = 0.30, p < 0.001; Fig S2), in contrast to previous studies that reported significant positive relationships between park size and vegetation or soil C stocks [65,66]. This weak relationship may reflect the high heterogeneity among urban parks. Some urban parks are intentionally designed as sports fields or open grasslands, where vegetation density is deliberately kept low for recreational or aesthetic purposes. This heterogeneity is also visually evident in the spatial distribution and proportional patterns of UGCI shown in Figs 3a and 3b, where substantial variation is observed among parks of similar size.

***9. The manuscript closes with generic advice: “create buffer zones”, “reduce soil compaction”, “apply multi-layer planting”. No cost estimates, no trade-off analyses, no references to municipal codes or land-opportunity prices (USD > 1 000 m2 in Suwon) are provided. Such recommendations are not actionable and fall well short of PLOS ONE’s requirement that papers offer “evidence-based, practical solutions”.***

Response: We appreciate the reviewer’s insightful comment. We acknowledge that our management suggestions remain general and do not encompass detailed strategic planning, such as cost-benefit analyses or policy implementation pathways. We believe that the economic evaluation of management strategies should be considered in future studies. To clarify our limitation and future direction, we have explicitly added this in Section 3.5 Limitation and future study.

- Revised Text (Lines 534-542): This study had several clear limitations that can be addressed in future research. First, the UGCI was currently focused on C enhancement and did not integrate policy implementation pathways or cost-benefit considerations. Because its primary role was to provide a scientifically grounded, spatially explicit diagnosis of low-carbon areas and management directions, economic assessments were intentionally kept external to the index. Future studies could link the UGCI with additional decision layers, such as cost-effectiveness, marginal abatement cost, life-cycle assessment, or budgeting, through multi-criteria decision analysis. This would enhance the applicability of the index while preserving flexibility to local financial capacities and policy priorities.

***Reviewer #3:***

***The results and discussion section is too weak to support the final conclusion in this manuscript. in contrast, the author put too many details on the materials/methods section, which make the structure of this manuscript is unbalanced. Also, all figures are in a limited quality. I don't think the current manuscript meet the standard of the submitted journal.***

Response: We sincerely appreciate your time and effort in reviewing our manuscript. Following the other reviewers’ constructive feedbacks—particularly regarding the lack of a cost–benefit analysis, the logical justification of the index, and data validation—we have made extensive revisions and believe the manuscript has improved considerably.

The main changes incorporated into the revised manuscript are summarized below:

- **Conceptual refinement of the index:** Following a critical suggestion from Reviewer #2, we have renamed the "Carbon Management Prioritizing Index (CMPI)" to the "Urban Green Carbon Index (UGCI)." This change more accurately reflects the index's function as a diagnostic tool for assessing carbon status, distinct from a full cost-benefit or opportunity-cost analysis, thereby clarifying the manuscript's core contribution.
- **Strengthened methodological justification:** We have provided more detailed explanations for key methodological decisions. This includes a clearer rationale for selecting the 30 m grid size, justification for the choice of input variables for our GPP/Reco models (as suggested by Reviewer #5), and a more transparent description of the data resampling and processing techniques (as requested by Reviewer #4).
- **Enhanced validation and contextualization:** To address comments regarding the validation of our models and the novelty of our index (Reviewers #2, #4, and #5), we have expanded our discussion. We now include a direct quantitative comparison of the UGCI with an existing metric, the Carbon Sequestration Potential Index (CSPI), to better situate our work within the current literature and highlight its unique contributions to urban carbon management.
- **Refinement of interpretations and claims:** Based on the feedback concerning potential logical fallacies (Reviewers #2 and #4), we have carefully revised our interpretations. For instance, we replaced ambiguous terms like "degraded" with more precise descriptions such as "under poor condition" and have moderated our claims where correlations were weak, ensuring our conclusions are robustly supported by the evidence presented.
- **Improved reproducibility and clarity:** In response to requests for greater detail (Reviewers #4 and #5), we have added specific parameters for equations, elaborated on model validation procedures, and moved detailed methodological descriptions to the supplementary materials to ensure clarity and reproducibility without disrupting the flow of the main text. The entire manuscript has also undergone a thorough proofread to improve language and readability.

We would be grateful for any further comments you could provide on the revised version.

**Reviewer #4:**

***This manuscript presents an interesting framework for a Carbon Management Priority Index (CMPI) that integrates vegetation, soil, and productivity indicators at fine resolution for urban carbon management. Several issues need to be addressed:***

***1. The manuscript relies on multi-resolution datasets that are all resampled to 30 m, yet the methods for resampling are insufficiently explained. Please clarify exactly how each dataset was resampled, justify the choices, and provide an assessment of how resampling errors may affect CMPI outputs.***

Response: We appreciate the reviewer’s comment. We clarified the resampling methods and justified the choice of the methods for each dataset in Section 2.2.1, 2.2.2 and 2.2.3.

- Original text:
  (Lines 214-216) Using this new fine-scale map, we extracted green areas and resampled them to a 30 m resolution by assigning the dominant land cover type in each grid.
  (Lines 261-262) To unify the spatial resolution with other datasets, we resampled the map to a 30 m resolution using bilinear interpolation method.
  (Lines 272-274) Net C uptake, which is represented by net ecosystem productivity (NEP), was calculated for each 30 m grid as the difference between gross primary productivity (GPP) and ecosystem respiration (Reco).
- Revised text:
  (Lines 215-216) Using the updated 1 m land-cover map, we aggregated it to a 30 m grid by assigning each grid cell the dominant land-cover type when multiple classes were present.
  (Lines 269-271) To unify the spatial resolution with other datasets, we resampled the map to a 30 m resolution using a bilinear interpolation method, preserving its continuous gradient.
  (Lines 274-277) Net C uptake, which is represented by net ecosystem productivity (NEP), was calculated for each 250 m grid as the difference between gross primary productivity (GPP) and ecosystem respiration (R_eco_). Then, the spatial resolution was downscaled to a 30 m grid basis using a bilinear interpolation method to preserve the continuous data gradient.

***2. The refinement of land-use data from 1 m to 0.25 m resolution also needs stronger justification (Line 201-210). Since the final analysis is conducted at 30 m, it is unclear whether this refinement adds meaningful information beyond additional processing. Please provide quantitative evidence that this improves classification accuracy and ultimately influences CMPI estimates.***

Response: We appreciate the reviewer’s comment regarding the refinement from 1 m to 0.25 m resolution. The refinement was essential for capturing highly fragmented urban vegetation, particularly narrow roadside and interstitial green strips that are easily undetected at coarser resolutions (>1 m). Performing the analysis at lower resolution often caused the edges of tree canopies to be misclassified or excluded from the roadside vegetation category when computing tree-canopy cover. Maintaining the higher available resolution therefore reduced such uncertainties and improved the assessment accuracy of fine-scale vegetation.

By integrating the U-Net derived fine scale roadside vegetation map with the existing 1 m subdivision land cover map, we identified an additional 13.69% of tree-covered area that was not represented previously, achieving a tree-classification accuracy of 96.29% (Supplementary Text S1). This refinement significantly enhances the accuracy of vegetation C storage assessment in complex and fragmented urban environments. We have added this detail in Section 2.2.1. Land cover classification.

- Original text (Lines 208-210): The model was developed using airborne LiDAR and RGB orthogonal imagery with a resolution of 0.25 m as input data for roadside vegetation classification, based on the U-Net algorithm [25].
- Revised text (Lines 202-211): Because this base map did not separately delineate urban parks and roadside vegetation, we refined it using supplementary datasets. Urban parks were identified from the park boundary data of the Korean Ministry of Land, Infrastructure and Transport, and the roadside vegetation was mapped using a deep-learning classification model based on the U-Net algorithm [25]. The U-Net model employed airborne LiDAR and high-resolution (0.25 m) RGB orthogonal imagery to detect tree-covered areas along roads. The model successfully identified an additional 13.69 % of tree-covered areas that had not been classified in previous datasets, achieving a classification accuracy of 96.29 % (Supplementary Text S1). This refinement was essential for accurately representing highly fragmented urban vegetation often overlooked at coarser resolutions (>1 m).

***3. In Section 2, the roadside vegetation correction model, the U-Net classification, and the digital soil mapping are described only briefly, without sufficient information on model type, input features, training and validation procedures, hyperparameters, or performance metrics. These details are critical for reproducibility and should be provided, at least in supplementary materials.***

Response: We thank the reviewer’s constructive comment. Admitting the reviewer’s suggestion, we have added the detailed methodology about land cover classification using U-Net algorithm, bias correction for vegetation C storage in supplementary text S1 and S2, respectively. The digital soil mapping procedures, hyperparameters and performance metrics were described in the main manuscript and Supplementary Table S1.

- Revised text (Lines 335-340): Three machine-learning algorithms—random forest, artificial neural network, and light gradient boosting—were evaluated using these predictors (Table 1). The performance of each model was assessed using a 10-fold cross-validation approach. The optimized hyperparameter by the Bayesian optimization method and the performance of optimized models were described in Table S1. The light gradient boosting model performed best, yielding an R² of 0.84 and an RMSE of 10.33%.

***4. The discussion does not sufficiently situate CMPI in relation to existing indices and prior studies. A more explicit comparison with other urban carbon assessment methods would help demonstrate what CMPI adds, both in terms of input variables, weighting strategies, and management relevance. Even a qualitative comparison, if quantitative replication is not feasible, would make the contribution more convincing.***

Response: We appreciate the reviewer’s valid comment. Following the reviewer’s suggestion, we have added the comparison methodology in Supplementary Text S4 ‘Comparison between UGCI and an existing index’ and in Section 3.4 ‘Comparison between UGCI and an existing carbon management index’ to the revised manuscript.

- Revised text:
  **Supplementary Text S4. Comparison between UGCI and an existing index**
  To examine the applicability of the UGCI in urban environments, we compared both the statistical and spatial distributions of the UGCI with those of the carbon sequestration potential index (CSPI) developed by Pascual et al. (2020). The CSPI was originally designed to identify suitable areas for afforestation and reforestation management across six Hawaiian Islands using 30 m resolution datasets. The calculation of the CSPI is as follows:
  $CSPI=GPP \times\left( ACD \right)^{-1}\times\left( 1-FC \right)$	(1)where GPP (gross primary production) (kgC m^-2^ yr^-1^), ACD (aboveground carbon density) (kgC m^-2^), and FC (Forest coverage) (%).

  We used the GPP (tC ha^-1^ yr^-1^) and vegetation C storage (tC ha^-1^) developed in this study to calculate the CSPI. To adapt the CSPI for an urban context, the forest coverage term in each grid was replaced with green-space coverage. After calculating the CSPI, the results were min-max normalized, following the same procedure used for UGCI visualization, to enable a direct comparison between CSPI and UGCI.

  (Lines 501 – 531) **Comparison between UGCI and an existing carbon management index**
  To evaluate whether the UGCI provides a more effective metric for urban C management than previously proposed index, we compared its statistical and spatial distributions with those of the Carbon Sequestration Potential Index (CSPI) developed by Pascual et al. (2020). The detailed methodology for calculating the CSPI is provided in Supplementary Text S5. The data distribution of the UGCI was relatively closer to a normal distribution (Fig S3), supporting its higher discriminative capacity for assessing C conditions within heterogeneous urban environments.

**Fig 7 Comparison between the Urban Green Carbon Index (UGCI) and the Carbon Sequestration Potential Index (CSPI).** Spatial distributions of (a) UGCI and (b) CSPI across the study area are shown. The red box marks the region of interest, for which detailed views are provided in (c) UGCI, and (d) CSPI.

Figs 7a and 7b illustrate the spatial distributions of UGCI and CSPI, respectively. Because the UGCI and CSPI were developed for different purposes, their spatial patterns across the urban landscape differed markedly. In the UGCI map, clear gradients were observed between forest edges and interiors within forest patches, whereas the CSPI did not capture such within-patch variations, showing near-zero values across forested areas. This is because the CSPI was designed to directly represent the potential only for afforestation management, thus assigning low potential to areas that are already forested. Unlike the CSPI, the UGCI captures both low-biomass areas requiring vegetation establishment and high-biomass forests with limited NEP or soil C condition, thereby identifying diverse C management needs across urban landscapes.

High CSPI values were mostly found in agricultural areas (Figs 7c and 7d), which reflects their low aboveground C density. However, because agricultural systems are managed to maintain intentionally low aboveground biomass compared to forests, the CSPI—constructed solely from vegetation-related components—has limited capability in evaluating the C status of non-forest green spaces. Considering the diverse land-cover types in urban green spaces, the UGCI’s integrated vegetation and soil scheme offers a more comprehensive representation of urban C storage and sequestration potential.

***5. Line428-429, the correlation reported between patch size and CMPI (r = –0.27, p < 0.001) is weak. This does not justify the strong claim that smaller patches “generally” had higher CMPI values.***

Response: We fully agree with the reviewer’s comment. As the correlation was too weak, we have described the reason for the weak correlation instead of highlighting the correlation between patch size and the index. The revised lines were as follows:

- Original text (Lines 428-434): Additionally, CMPI was inversely related to patch size in urban parks, indicating smaller patches generally had higher CMPI values (r = -0.27, p < 0.001; Fig. 6). Larger patches inherently support more complex ecological structures, higher vegetation density, and greater soil C inputs, leading to improved C sequestration capacity [65–68]. Thus, CMPI effectively reflects the ecological and landscape characteristics that influence urban green spaces’ C sequestration potential. Overall, our results confirm that CMPI is a robust diagnostic tool for identifying and prioritizing urban green spaces requiring targeted management interventions.
- Revised text (Lines 447-455): Additionally, the correlation between the areas of green patches in urban parks (logarithmic scale) and their mean UGCI was very weak (r = 0.30, p < 0.001; Fig S2), in contrast to previous studies that reported significant positive relationships between park size and vegetation or soil C stocks [65,66]. This weak relationship may reflect the high heterogeneity among urban parks. Some urban parks are intentionally designed as sports fields or open grasslands, where vegetation density is deliberately kept low for recreational or aesthetic purposes. This heterogeneity is also visually evident in the spatial distribution and proportional patterns of UGCI shown in Figs 3a and 3b, where substantial variation is observed among parks of similar size.

***Reviewer #5:***

***Dear Authors,***

***I have read carefully the manuscript entitled "A Comprehensive Decision-Making Framework for Prioritizing Carbon Management in Complex Urban Landscape" submitted by Seo et al. to PLOS One.

This manuscript seeks to introduce a novel decision-making framework designed to evaluate existing carbon storage and sequestration and to identify areas exhibiting deteriorated carbon dynamics.

Based on my review, I find that this manuscript addresses a highly relevant topic, given the urgent need for carbon neutrality strategies in urban areas. The proposed framework offers a practical and scientifically sound tool to support public policies for carbon management in urban landscapes. However, several issues require careful attention before the manuscript can be considered for publication. The key points are outlined below:***

 Response: We are very grateful for your positive and encouraging review. Thank you for the thoughtful and constructive feedback; your suggestions have been invaluable in strengthening the manuscript. We have revised the article to incorporate your recommendations, and you will find my detailed responses to each of your points below.
 ***1) MATERIALS AND METHODS

1.1 The authors use EVI, LSWI, PAR, Tair, and VPD, but they do not discuss why these indices were chosen instead of others (e.g., NDVI, drought index, soil moisture).***

Response: We appreciate this comment. Our predictors follow the Vegetation Photosynthesis and Respiration Model (VPRM) framework developed by Mahadevan et al. (2008), which adopts a deliberately simple parameterization to capture the dominant controls of GEE and NEE while maintaining robustness for inversion at fine spatiotemporal scales. In VPRM, EVI is used instead of NDVI because it is more closely correlated with photosynthesis across a wider LAI range and is less prone to saturation; it also leverages the blue band to reduce atmospheric and soil‑background effects. LSWI (NIR–SWIR) is employed to characterize canopy water status and phenology, which enter the model as multiplicative scalars on light‑use efficiency together with air temperature (Tair). PAR provides the light‑dependent driver of GEE.

Regarding soil moisture and drought indices, widely used satellite soil‑moisture products (e.g., SMAP) have typical grid spacings on the order of 9–36 km, which are substantially coarser than the urban context of our study, and can lead to scale mismatch; therefore we relied on LSWI to reflect canopy‑level water conditions consistently across sites. We additionally included VPD to explicitly represent atmospheric dryness, a well‑established constraint on stomatal conductance and photosynthesis across biomes; incorporating VPD is consistent with subsequent developments and evaluations of VPRM‑type models. We have added text in Section 2.2.3 to clarify these choices and to cite the original framework.

- Original text (Lines 276-280): The key input variables for this model included air temperature and vapor pressure deficit (VPD) from Local Data Assimilation and Prediction System (LDAPS) provided by Korea Meteorological Administration, photosynthetically active radiation (PAR) from HIMAWARI-8, and enhanced vegetation index (EVI) and land surface water index (LSWI) from MODIS product.
- Revised text (Lines 279-286): The model structure and choice of predictors followed the vegetation photosynthesis and respiration model, which provides a simple yet robust parameterization of gross ecosystem exchange and net ecosystem exchange through the use of enhanced vegetation index (EVI), land surface water index (LSWI), and meteorological scalars [35]. The key input variables for this model included air temperature and vapor pressure deficit (VPD) from the Local Data Assimilation and Prediction System (LDAPS) provided by Korea Meteorological Administration, photosynthetically active radiation (PAR) from HIMAWARI-8, and EVI and LSWI from MODIS product.

***1.2 In the Reco equation, the parameters a, b, and c are introduced, but their values, fitting ranges, or calibration methods are not provided. This omission limits the reproducibility of the study.***

Response: We thank the reviewer for pointing this out. Following the reviewer’s suggestion, we added the exact values of the introduced parameters in the equation and described the detailed methodology, including fitting ranges and calibration methods, in Supplementary text S3. We clarified this in the text briefly as well.

- Original text (Lines 294-299):
  Ecosystem respiration (Reco) was modeled as a function of the GPP accumulated during the 7 days preceding a specific date (GPPacc) and the air temperature (Tair), reflecting the impact of vegetation productivity on respiration rates [37,38]:

$R_{eco}=(a+b\times{GPP}_{acc})\times exp(c\times\frac{T_{air}}{\max\left( T_{air} \right)})$	(5)

where a, b, and c are constants, GPPacc is the accumulated GPP (μmol CO_2_ m⁻²), and Tair is the air temperature (℃).

- Revised text (Lines 304-312):
  Hourly ecosystem respiration (Reco) was modeled as a function of the GPP accumulated during the 7 days preceding a specific date (GPPacc) and the air temperature (Tair), reflecting the impact of vegetation productivity on respiration rates (Eq 10) [37,38]. The model parameters were initialized within the empirical ranges suggested by the existing studies [35,38] and subsequently optimized using the FLUXNET2015 data and meteorological data to minimize the residuals between modelled and observed Reco.

$R_{eco}=(1+0.20\times{GPP}_{acc})\times exp(1.16\times\frac{T_{air}}{\max\left( T_{air} \right)})$	(10)

where Reco is the ecosystem respiration (μmol CO_2_ m^-2^ s^-1^), GPPacc is the accumulated GPP (μmol CO_2_ m^-2^ s^-1^), and Tair is the air temperature (℃).

***1.3 The reported Rˆ2 = 0.76 indicates a reasonably good model fit, but it is not particularly strong. The authors do not discuss the limitations of the model, such as potential biases related to seasonality, differences among vegetation types, or the influence of extreme events.***

Response: We agree that an R^2^ of 0.76 is reasonable but not exceptional, and we have explicitly discussed model limitations in Section 3.5. Limitation and future study.

- Revised text (Lines 547-554): Lastly, the reliability of UGCI analysis depends on the availability of fine-scale datasets and validation through field observations. Because grid-based analysis involves resampling multiple datasets, collecting the finest original data is critical. Although this study performed validation using available field data, the limiting number of observation system (e.g., eddy covariance tower) may introduce biases related to seasonality, vegetation composition, and extreme climate events. Continued development of high-resolution datasets and the expansion of observation networks across diverse land-cover types will be essential for improving the accuracy and generalization of UGCI-based assessments.

***1.4 Although the authors indicate the units for GPP, they do not do the same for Reco in the corresponding formula.***

Response: We appreciate the reviewer’s comment. We have added the unit of Reco on Eq 10.

- Revised text (Lines 310-312):

$R_{eco}=(1+0.20\times{GPP}_{acc})\times exp(1.16\times\frac{T_{air}}{\max\left( T_{air} \right)})$	(10)

where Reco is the ecosystem respiration (μmol CO2 m-2 s-1), GPPacc is the accumulated GPP (μmol CO2 m-2 s-1), and Tair is the air temperature (℃).

***1.5 It is only implied, but not clearly stated, whether the calculation is performed on a daily or hourly basis.***

Response: We appreciate the reviewer’s comment. We have clarified the temporal scale of model calculation as follows:

- Original text:
  (Lines 275-276) GPP was estimated using a random forest model based on the light-use efficiency (LUE) concept [35].
  (Lines 294-296) Ecosystem respiration (Reco) was modeled as a function of the GPP accumulated during the 7 days preceding a specific date (GPPacc) and the air temperature (Tair), reflecting the impact of vegetation productivity on respiration rates [37,38]
- Revised text:
  (Lines 278-279) GPP was estimated using a random forest model based on the light-use efficiency (LUE) concept on a daily basis [35].

(Lines 304-309) Hourly ecosystem respiration (Reco) was modeled as a function of the GPP accumulated during the 7 days preceding a specific date (GPPacc) and the air temperature (Tair), reflecting the impact of vegetation productivity on respiration rates (Eq 10) [37,38]. The model parameters were initialized within the empirical ranges suggested by the existing studies [35,38] and subsequently optimized using the FLUXNET2015 data and meteorological data to minimize the residuals between modelled and observed Reco.

***1.6 Validation using only one flux tower (Mt. Taehwa) may introduce bias. Consequently, the model may not generalize well across different land-cover types.***

Response: We appreciate the reviewer’s comment, which is similar to the point raised by Reviewer 2 regarding the potential bias caused by validating the model using only the Mt. Taehwa flux tower. As explained in our response to Reviewer 2 (Comment 5), Mt. Taehwa was the only long-term eddy-covariance dataset available near the study area, and therefore was used for validation. Importantly, our index approach relies on spatially normalized NEP, for which capturing spatial variability is more critical than reproducing the absolute fluxes. The temporal patterns of GPP and Reco observed at Mt. Taehwa were also consistent with those recorded at recently established urban-forest eddy-covariance towers (e.g. Mt. Nam and Changgyeong Palace in Seoul), supporting the representativeness of the selected site.

We have clarified this explanation in Section 2.2.3 and noted in Section 3.5 Limitation and future study that, through the ongoing project, additional flux-tower observations are being implemented across diverse urban and peri-urban land-cover types to further improve the generalization of NEP estimation models.

- Original text:
  (Lines 291-293) The GPP model was trained using the FLUXNET2015 dataset [36] and validated against local flux tower measurements at Mt. Taehwa in South Korea (37°18'15" N, 127°18'50" E), achieving a R^2^ of 0.76 and a root mean square error (RMSE) of 0.41 μmol CO_2_ m^-2^ s^-1^.
- Revised text:
  (Lines 298-303) The GPP model was trained using the FLUXNET2015 dataset [36] and validated against local flux tower measurements at Mt. Taehwa in South Korea (37°18'15" N, 127°18'50" E), achieving an R^2^ of 0.76 and an RMSE of 0.41 μmol CO_2_ m^-2^ s^-1^. Although Mt. Taehwa is located outside our study site, the temporal variation patterns of its GPP and Reco data were consistent with those observed at the eddy covariance towers in Mt. Nam and Changgyeong Palace, representative urban green areas in Seoul.
  (Lines 547-554) Lastly, the reliability of UGCI analysis depends on the availability of fine-scale datasets and validation through field observations. Because grid-based analysis involves resampling multiple datasets, the quality of the original data resolution is critical. Although this study performed validation using available field data, the limiting number of observation system (e.g., eddy covariance tower) may introduce biases related to seasonality, vegetation composition, and extreme climate events. Continued development of high-resolution datasets and the expansion of observation networks across diverse urban and peri-urban land-cover types will be essential for improving the accuracy and generalization of UGCI-based assessments.

***1.7 The authors defined 30 m as the threshold, but they did not justify this choice. It is unclear whether this value was based on previous literature, the resolution of the datasets, or a methodological decision.***

Response: We appreciate the reviewer’s valuable comment. The 30 m threshold was determined as the finest available resolution among the input datasets and was used as the reference scale for downscaling coarser layers. This setting allowed us to represent the high spatial heterogeneity of urban environments while remaining consistent with the practical limits of data availability. Although a 1 m land-cover map was available for vegetation carbon storage, the other components lacked data at such a fine scale. Therefore, a 30 m grid was adopted as the optimal compromise between spatial detail and dataset consistency. We have added this explanation to Section 2.2 Case Study to clarify the rationale for selecting a 30 m grid.

- Revised text (Lines 190-193): In this case study, the data collection was conducted for the year 2020. All spatial data layers were resampled to a 30 m resolution, balancing the finest available resolution of input datasets with the spatial granularity required for urban-scale analysis.

***2) RESULTS AND DISCUSSION

2.1 Figure 3c shows NEP ranging from –0.69 to 16.95 t C ha-1 yr-1. Could the authors clarify the negative value?***

Response: We thank the reviewer for pointing this out. Negative NEP values indicate that ecosystem respiration exceeded gross primary productivity, meaning that the ecosystem acted as a net carbon source rather than a sink in 2020. In our dataset, this occurred mainly in roadside vegetation near the city center, where environmental stressors such as urban heat island and extremely dry soils can suppress photosynthesis and enhance ecosystem respiration. We have clarified this point more explicitly in Section 3.1.

- Original text (Lines 365-369): Roadside vegetation displayed the lowest values, including net C emissions, particularly near the city center. This result suggests environmental stressors in central urban areas, such as heat wave during summer or extremely dry soil during winter, are deteriorating roadside vegetation C storage and sequestration [50–52].
- Revised text (Lines 386-390):
  Roadside vegetation displayed the lowest values, including net C emissions (negative NEP values), particularly near the city center. This result suggests environmental stressors in central urban areas, such as heat wave during summer or extremely dry soil during winter, are deteriorating roadside vegetation C storage and sequestration [50–52].

***2.2 The authors state that CMPI is “more effective” than previous approaches, but the comparison appears mainly descriptive. Could the authors provide a quantitative comparison or benchmarking against existing indices?***
 Response: We appreciate the reviewer’s valuable comment. Following the reviewer’s suggestion, we have added a quantitative comparison with an existing index to evaluate the performance of the proposed index. Specifically, Supplementary Text S4 ‘Comparison between UGCI and an existing index’ now describes the calculation method of the Carbon Sequestration Potential Index (CSPI; Pascual et al., 2020), and Section 3.4 ‘Comparison between UGCI and an existing carbon management index’ presents the results of spatial and statistical comparisons between UGCI and CSPI. These additions allow for a more quantitative benchmarking of our index against existing approaches (Lines 499-529).

***2.3 How do the authors assess the propagation of uncertainties from the GSOC data and the GPP/Reco modeling into the CMPI values?***
 Response: We appreciate the reviewer’s insightful comment regarding uncertainty propagation. The UGCI is a relative, normalized index, in which all components were first scaled to a 0-1 range and then integrated through a weighted linear formulation. This structure substantially reduces the influence of absolute errors; therefore, input uncertainties affect the relative ranking of grids rather than their absolute magnitudes.

To evaluate potential uncertainty propagation, using a one-at-a-time approach, where each component (vegetation C storage, soil C storage, NEP, and silt–clay content) was perturbed individually by its RMSE while the others were held constant. This method allows us to isolate the effect of each input on the UGCI’s spatial pattern. Because the UGCI is a normalized, weighted-linear index, the propagation effects from multiple components are largely additive; therefore, a single-factor perturbation adequately represents the overall uncertainty behavior.

When each variable was perturbed by its RMSE, the spatial correlation between the baseline and perturbed UGCI maps remained above 0.9 (0.999 for vegetation C storage, 0.985 for SOC, 0.995 for NEP, and 0.999 in silt-clay), indicating that uncertainty propagation had only a minor effect on the overall spatial pattern of the index.

***2.4 The results represent a specific time period, but urban carbon dynamics can vary under extreme climate events. Do the authors plan to validate the CMPI using longer time series or under climate change scenarios?***

Response: We appreciate the reviewer’s constructive comment. The UGCI was designed as a framework to propose management suggestions based on the current C status of urban green spaces, representing the first step toward dynamic monitoring of C sequestration. We fully acknowledge that the present analysis is limited to a single reference period and does not yet capture temporal variations or extreme climate events. We are aware that the practical relevance of this framework depends on its ability to account for temporal dynamics and to remain robust under climate extremes.

To address this, a follow-up project led by the same research team is currently producing the datasets at six-month intervals. Incorporating these sub-real-time datasets into the UGCI framework will allow us to simulate the temporal dynamics of UGCI and assess its response to extreme climate events. We have added this statement in Section 3.5 Limitation and future study, emphasizing that verifying UGCI variability under extreme conditions and management interventions will be a key direction for future work.

- Revised text (Lines 543-547): Second, the UGCI framework did not explicitly account for ecological interactions or nonlinear feedback among its constituent components. Once these interrelationships are quantitatively understood, incorporating them into the index could improve its ability to represent complex ecosystem processes and capture spatiotemporal variability in C dynamics.
