# Supplementary material for: The urban green carbon index (UGCI): A spatial framework for suggesting urban carbon management
Source: PLoS One. 2026 May 12;21(5):e0347782. doi: 10.1371/journal.pone.0347782 (PMC13166916; doi:10.1371/journal.pone.0347782)
Supplement: Attachment — Submitted filename: Response to reviewer_UGCI_final_2026.docx [file pone.0347782.s004.docx]

**Response to Reviewers [PONE-D-25-21587R1]**

Dear Dr. Tianheng Shu, Academic editor:

Thank you for the opportunity to revise our manuscript, ID: PONE-D-25-21587R1, titled “The urban green carbon index (UGCI): A spatial framework for suggesting urban carbon management.” We sincerely appreciate your guidance and the reviewers’ thoughtful comments throughout the review process. We have carefully addressed the additional comments from the reviewer and refined the manuscript accordingly.

The main changes incorporated into the revised manuscript are summarized below:

- **Clarified the use of soil texture as a proxy indicator:** In response to the suggestion from Reviewer #2, we explicitly state that soil silt and clay content is used as a feasible proxy for physical carbon stabilization potential. We also added a forward-looking note that UGCI could incorporate direct measures of soil carbon saturation deficit or stabilization capacity when spatially explicit datasets become available at relevant scales.
- **Expanded limitations and future directions for NEP validation:** We expanded Section 3.5 to more clearly acknowledge the limitation of validating NEP estimates using a single flux tower and the uncertainty associated with extrapolating to diverse urban land covers. We further suggested that future work should establish additional micrometeorological and/or chamber-based flux measurements across distinct urban green space types to directly calibrate/validate carbon flux model estimates, thereby reducing uncertainty in the NEP component of the UGCI.
- **Added more specific illustrative examples to management strategies:** According to the suggestion from Reviewer #2, we refined the management recommendations in Section 3.3 by integrating brief, concrete examples into key strategies. This improves practical clarity while maintaining the science-driven diagnostic focus without introducing a full cost analysis.

Here, we provide responses following the comments by the reviewers.

**Reviewer #1:**

***The authors have well addressed our comments and improved the quality of this manuscript. I have no more technical comments.***

Response: We sincerely thank the reviewer for the positive evaluation and for confirming that no further technical comments are needed. The reviewer’s previous suggestions were very helpful in improving the manuscript’s clarity and presentation. We appreciate the reviewer’s careful assessment, which has significantly contributed to enhancing the quality of the manuscript.

***Reviewer #2:***

***This study presents a novel, spatially explicit decision-support framework that integrates vegetation carbon storage, soil carbon storage, net carbon uptake, and soil carbon storage potential into a composite “Urban Green Carbon Index (UGCI)”. Coupled with ternary plot analysis, the framework provides a diagnostic tool and a basis for prescribing site-specific management strategies for urban carbon sequestration. The research addresses a scientifically significant and policy-relevant topic. The methodological approach is robust, combining high-resolution remote sensing, modeling, and machine learning techniques. The case study in Suwon, South Korea, is well-designed, and the results are presented and analyzed thoroughly. The authors have undertaken extensive and thoughtful revisions in response to the reviewers’ comments. Key improvements include the conceptual refinement of the index (renaming from CMPI to UGCI), strengthened methodological justifications, enhanced validation and contextualization through comparison with an existing index, more nuanced interpretation of results, and clearer acknowledgment of limitations. These revisions have significantly strengthened the manuscript's clarity, rigor, and impact.***

***While the manuscript is of high quality, the following minor points could further enhance it:***

Response: We sincerely thank the reviewer for the detailed and positive assessment of our revised manuscript. We are particularly grateful for the reviewer’s earlier in-depth critiques, which greatly improved the conceptual clarity, scientific rigor, and policy relevance of the study. The manuscript has been revised to incorporate the reviewer’s suggestions, which we believe have further improved the clarity of our article. Please find our detailed responses to each of your comments outlined below.

***1. In Section 2.2.4 or the Discussion/Limitations, consider adding a sentence explicitly stating that soil texture is used as a feasible proxy indicator for physical carbon stabilization potential, acknowledging that future iterations of UGCI could incorporate more direct measures of soil carbon saturation deficit or stabilization capacity as such data become available at relevant scales.***

Response: We appreciate the reviewer’s constructive suggestion. We have clarified that silt and clay content is used as a feasible proxy indicator of physical C stabilization potential, and could be updated with other direct measures if the spatially explicit data become available at relevant scales. These points have been incorporated into Section 2.2.4 Soil C storage potential: Silt and clay content.

- Revised Text (Lines 323-330): Given that soil silt and clay content play a critical role in stabilizing C [39,40], we used silt and clay content as a feasible proxy indicator of physical C storage potential. If a more direct indicator for C storage potential, such as soil C saturation deficit or stabilization capacity, becomes available at a spatially relevant scale, the soil C storage potential component of UGCI could be updated to incorporate those direct metrics. While silt and clay content is intrinsic and relatively stable in natural soils, urban soils—particularly those in parks and roadside vegetation—are often reconstructed and sand-dominated, allowing urban soil texture to be modified during construction.

***2.The authors correctly acknowledge the limitation of using a single flux tower for model validation and extrapolation to diverse urban interiors. In Section 3.5, this point could be slightly expanded. It could be suggested that future work aims to establish more micrometeorological or chamber-based flux measurements across distinct urban green space types (e.g., pocket parks, green roofs) to directly calibrate/validate carbon flux models for the specific urban matrix, thereby reducing uncertainty in the NEP component of UGCI.***

Response: We thank the reviewer for this helpful recommendation. In Section 3.5 of our manuscript, we have suggested that uncertainty in the net C uptake of UGCI could be reduced by establishing additional observations across distinct urban green space types to directly calibrate/validate model estimates.

- Revised Text (Lines 556-567): Lastly, the reliability of UGCI analysis depends on the availability of fine-scale datasets and validation through field observations. Because grid-based analysis involves resampling multiple datasets, collecting the finest original data is critical. Although this study performed validation using available field data, the limiting number of observation system (e.g., eddy covariance tower) may introduce biases related to seasonality, vegetation composition, and extreme climate events. To reduce uncertainty in the net C uptake of UGCI, future work should expand validation efforts by establishing additional micrometeorological measurements and chamber-based flux observations across distinct urban green space types (e.g., pocket parks, roadside plants, and green roofs), enabling direct calibration and validation of the model estimations. Continued development of high-resolution datasets and the expansion of observation networks across diverse land-cover types will be essential for improving the accuracy and generalization of UGCI-based assessments.

***3. In Section 3.3, when listing strategies like “reducing soil compaction” or “multi-layered planting”, you could add a brief illustrative example without delving into full cost analysis. For instance: “...such as using organic mulch amendments and planting deep-rooted species to alleviate compaction in urban parks”, or “...prioritizing drought-tolerant understory species in multi-layered roadside plantings”. This adds specificity while maintaining the focus on science-driven diagnosis.***

***This is a well-executed study with strong conceptual and methodological contributions to the field of urban carbon management. The authors have successfully addressed the substantive concerns raised during review. The manuscript is now suitable for publication, pending incorporation of the minor, constructive suggestions outlined above.***

Response: We appreciate the reviewer’s constructive suggestion. We agree that adding brief but illustrative details can improve the practical clarity of the strategies. Therefore, we revised Section 3.3 to embed concise examples as follows:

- Revised Text (Lines 470-500): In forests, all grids classified as ‘low’ UGCI were spatially situated at edge areas, emphasizing the need for edge-focused management. These edge grids consistently exhibited the lowest scores for soil C storage, indicating that management efforts should primarily focus on enhancing soil C stability. Because forest-edge soils are more exposed to higher temperatures and direct sunlight, leading to accelerated decomposition [53,67], establishing shade-tolerant native understory shrubs/groundcovers and avoiding intensive leaf litter removal in edge areas can help minimize soil exposure and sustain soil moisture.

For agricultural areas, data points predominantly clustered near the upper vertex of the ternary plot, indicating limitations in both vegetation and soil C storage due to conventional farming practices. Here, management strategies should prioritize soil-focused measures, specifically adopting conservation agriculture practices such as retaining crop residues as surface mulch, reducing tillage intensity by shifting toward no-till or strip-till systems, and integrating biochar and compost applications to bolster soil C storage [71,72].

Urban parks exhibited management needs concentrated around soil C storage, largely driven by factors unique to urban green spaces, such as soil compaction and limited organic inputs. Therefore, targeted interventions that alleviate compaction through periodic aeration combined with organic mulch or compost amendments, preserve litter layers, and multilayered plantings using deep-rooted shrub species can effectively enhance soil C storage while supporting vegetation C storage simultaneously [73,74]. In addition, enhancing connectivity by linking parks through green corridors and adding small complementary green spaces such as pocket parks and vegetated street nodes as stepping stones can increase effective patches and strengthen C storage and sequestration in urban parks [64].

In contrast, roadside vegetation displayed more complex, multi-dimensional poor C patterns, with points frequently appearing near the ternary plot's center, suggesting concurrent poor condition in all three UGCI components. Effective management strategies in roadside vegetation should therefore integrate diverse practices, including designing multilayered roadside plantings that incorporate drought-tolerant understory species to increase vegetation C and their sustainability. Applying pruning regimes that maintain canopy vigor and sustained growth while avoiding excessive biomass removal, and mitigating soil sealing and compaction by enlarging planting strips and using permeable surfaces can be feasible to improve vegetation and soil C status.

***Reviewer #3:***

***The authors addressed most of the main comments from previous reviewers. I believe it makes the manuscript much better and is suitable for getting a publishment.***

Response: We greatly appreciate the reviewer’s thoughtful feedback and are pleased that the revised manuscript is now considered suitable for publication. The reviewer’s earlier comments were instrumental in improving the balance and strength of the manuscript. We thank the reviewer again for the constructive guidance that helped us substantially improve the manuscript.

**Reviewer #4:**

***The authors have responded adequately and revised the manuscript accordingly. I have no further major concerns.***

Response: We thank the reviewer for the encouraging comment and for confirming that there are no further major concerns. The reviewer’s earlier feedback was especially valuable for enhancing methodological transparency and reproducibility. We are grateful for the reviewer’s careful review, which significantly improved the rigor and clarity of the manuscript.

***Reviewer #5:***

***Dear Authors,***

***I have carefully read again the manuscript entitled "The Urban Green Carbon Index (UGCI): a spatial framework for suggesting urban carbon management" submitted by Seo et al. to PLOS One.***

***This manuscript seeks to introduce a novel decision-making framework designed to evaluate existing carbon storage and sequestration and to identify areas exhibiting deteriorated carbon dynamics.***

***Based on my review, I am satisfied with the authors’ responses to my comments.***

 Response: We sincerely thank the reviewer for the careful re-evaluation of our revised manuscript and for noting that our responses have adequately addressed the previous concerns. The reviewer’s earlier comments were highly valuable for improving the methodological justification, reporting completeness, and discussion of limitations. We appreciate the reviewer’s constructive feedback, which helped strengthen the overall quality and robustness of the manuscript.
